# Language Contact in Bilingual Brains: Formal Features in the Mental Representation of English–Spanish Bilingual Children

**DOI:** 10.3390/bs16060923

**Published:** 2026-06-04

**Authors:** Tamara Gómez Carrero, Raquel Fernández Fuertes

**Affiliations:** UVALAL, Departamento de Filología Inglesa, Universidad de Valladolid, 47011 Valladolid, Spain; raquelff@uva.es

**Keywords:** linguistic codeswitching, Spanish grammatical gender features, eye tracking during reading, language processing, child bilingual data, English-Spanish bilinguals

## Abstract

We combine formal linguistic approaches, where formal features are at stake, and psycholinguistic approaches to explore the processing mechanisms involved in language coactivation. In particular, we use linguistic codeswitching as a laboratory condition where the two languages of the bilingual are forced to be activated in order to explore how the properties of the two languages interact. We take English–Spanish switched determiner phrases (i.e., a switch between the determiner and the noun) to formally explore the directionality of the switch (*la/el* house vs. the *casa*) and the gender agreement mechanisms in Spanish determiner switches (*la* house vs. *la* book). We have elicited data via an eye tracking during reading experiment from simultaneous English–Spanish bilingual children who come from two language-contact contexts (Gibraltar and Sotogrande) and who have been grouped in terms of their language dominance: balanced and English-dominant bilinguals. Data have been analyzed using the first fixation duration measure on the noun and the regressions to the determiner. Results show that regressions to the English determiner are significantly higher than those to the Spanish determiner. This may suggest an economy of derivation as gender features need not be valued, something that would entail a delay in processing. The same patterns have been found in both bilingual groups, pointing to the necessity to tone down the importance of language dominance in language processing.

## 1. Introduction

The bilingual speaker has been placed at the center of language research to investigate language activation, how activation is modulated by control processes such as inhibition, and how language competition is resolved in both unilingual and bilingual settings (e.g., Abutalebi & Green, 2007; Bialystok et al., 2008; Declerck & Philipp, 2015; Kroll et al., 2012). As the bilingual has the special ability to switch between languages, extensive psycho- and neurolinguistic research has tried to understand the diverse cognitive mechanisms involved in language switches when using isolated item paradigms (e.g., Costa et al., 2006; Declerck & Philipp, 2015; Heikoop et al., 2016; Gollan & Ferreira, 2009; Meuter & Allport, 1999) and, more recently, with switched sentences where the two languages are in context, i.e., codeswitching (e.g., Byers-Heinlein et al., 2017; Fairchild & Van Hell, 2017; Fernández Fuertes et al., 2019, 2025; Van Hell et al., 2015, 2018).

Codeswitching, the alternation of the two languages within the same discourse, has prominently been explored from sociolinguistic and formal linguistics approaches during the last few decades. Mostly, it has been investigated as a language-in-contact phenomenon, taking into consideration the social situations in which it arises or the norms that govern its production (e.g., MacSwan, 1999, 2000; Muysken, 2000; Myers-Scotton, 1993; Poplack, 1980; Toribio, 2011). Additionally, codeswitching can be used as a linguistic context where the two languages are in contact and are simultaneously activated in the same structure, that is, linguistic codeswitching. Taking linguistic codeswitching as a tool, researchers aim to understand its cognitive and neural underpinnings (e.g., Byers-Heinlein et al., 2017; Gross et al., 2019; Valdés Kroff & Dussias, 2023; Van Hell et al., 2015, 2018), as well as how the properties of the two languages interact in the bilingual mind and the cognitive mechanisms that this interaction entails (e.g., Fairchild & Van Hell, 2017; Fernández Fuertes et al., 2019, 2025).

Taking into consideration the combination of theoretical and psycholinguistic approaches, where cognitive processes and formal features^1^ go hand in hand, we draw on linguistic codeswitching as an optimal context to explore how the properties of the two languages are represented in the mind of the bilingual and whether and how these mental representations may guide the cognitive processes which arise when the two languages of the bilingual are activated. In particular, we use English–Spanish linguistic codeswitching between a functional category, the determiner, and a lexical category, the noun, as in (1), where two directionalities are possible: an English determiner followed by a Spanish noun (1a), or a Spanish determiner followed by an English noun (1b).
1.a. The *libro* b. *El/La* book [the book]English determiner + Spanish noun Spanish determiner + English noun2.a. *El*_Masc_ book _SP Masc_/*La*_Fem_ house _SP Fem_ b. *La*_Fem_ book _SP Masc_/*El*_Masc_ house _SP Fem_ c. *El*_Masc default_ house _SP Fem_ [the book/the house]Gender congruent Gender non-congruent Masculine as default

In (1a), where English provides the determiner category, no gender agreement mechanisms are enforced between the English determiner and the Spanish noun, as English has no grammatical gender. In (1b), as seen below in (2), gender agreement between the Spanish determiner (*el/la*) and the English noun could be enforced. In this case, agreement would be established with the Spanish translation equivalent of the English noun (book > *libro*, house > *casa*). When this occurs, as in (2), the following grammatical gender mechanisms may result: gender congruent switches, where the gender of the determiner matches the gender of the Spanish translation equivalent of the English noun (2a); gender non-congruent switches, where no matching occurs (2b); or masculine as default switches, where Spanish grammatical gender is sub-specified for gender, and the gender of the determiner is set in masculine as a default option, regardless of the gender of the noun (2c).

In the present study, we aim to explore the cognitive mechanisms involved in structures where the two languages are forced to be activated at the same time, i.e., linguistic codeswitching, to understand how the grammatical properties of both English and Spanish can shape the processing of these switches. We do so by eliciting data from children, a population that has not been so much explored in this respect, and from two communities that are understudied. The English–Spanish bilingual children come from two particular language-contact contexts where codeswitching is a habitual practice in communicative interactions: Gibraltar, UK, and Sotogrande, Spain. Children have been grouped into balanced and English-dominant bilinguals according to their language dominance as per their Bilingual Language Profile scores (BLP, Birdsong et al., 2012). We formally explore the directionality of the switch (1 vs. 2) and the gender agreement mechanisms in Spanish determiner switches (2a vs. 2b vs. 2c) with an eye tracking during reading experiment.

## 2. Processing Mechanisms in Bilinguals

### 2.1. The Interaction Between the Two Languages of the Bilingual

The bilingual speaker has been shown to have a certain degree of simultaneous activation and interaction of their two languages, even in situations that are totally driven by just one language (e.g., Bialystok et al., 2012; Kootstra, 2015; Kroll et al., 2012). This has also been shown to be true when the characteristics of the two languages differ, i.e., different written scripts (e.g., Hoshino & Kroll, 2008) or when one of the two languages is signed (e.g., Morford et al., 2011). This means that the two languages of the bilingual are activated at the same time and that they can never be turned off, although they can be “turned down” (Tokowicz & Perfetti, 2005, p. 174).

Different models have been proposed for both comprehension (the bilingual interactive activation model, BIA (Grainger & Dijkstra, 1992) and its variants BIA+ (Dijkstra & van Heuven, 2002) or BIA-d (Grainger et al., 2010)) and production (the inhibitory control model, ICM, (Green, 1998)). These models attempt to explain how the modulation of language activation takes place as well as the different processes involved in this activation. In the case of the comprehension model, the modulation of activation is driven by bottom-up processes within the language system; that is, they are not external to the languages involved but are automatic processes internal to these languages. On the other hand, the production model requires domain-general processes outside the language system; that is, they are not specific to the languages involved.

Regardless of whether comprehension or production are at stake, language competition involves cognitive control mechanisms such as inhibition, i.e., suppressing or “turning down” one of the two languages. For the bilingual to inhibit one of their two languages has been argued to involve processing costs, which are also present when moving from one language to the other. This has been shown to be especially true in adult bilingual production (e.g., Costa et al., 2006; Heikoop et al., 2016; Gollan & Ferreira, 2009; Meuter & Allport, 1999; Olson, 2016). Processing costs have commonly been declared as asymmetrical when it comes to directionality (i.e., switching from language A into language B or from B into A); that is, it is more costly to move from the non-dominant language into the dominant language (e.g., Gou et al., 2011; Meuter & Allport, 1999). In the case of comprehension, processing costs have also been observed among bilingual children and adults (e.g., Byers-Heinlein et al., 2017; Fernández Fuertes et al., 2019, 2025; Gross et al., 2019; Olson, 2016; Potter et al., 2019). However, these processing costs have been shown to vary across tasks and across bilinguals’ profiles (e.g., Aparicio & Lavaur, 2014; Declerck & Grainger, 2017).

When it comes to exploring cognitive mechanisms across the two languages of the bilingual, two main approaches have been followed to deal with language alternation: switching across individual unrelated items and switching within a linguistic context. In the first case, studies have used paradigms that include unrelated items (i.e., single items which are switched between languages, such as the cued language switching paradigm or the alternating language paradigm, among others) (Van Hell et al., 2015) (for a review, see Bobb & Wodniecka, 2013 or Declerck & Philipp, 2015). More recently, in the second case, studies have used the language alternation that can appear as part of the bilingual repertoire, where the switch is in context. In this case, the focus has been placed on language competition and the resulting cognitive mechanisms involved, since both languages of the bilingual are active within the same structure (e.g., Byers-Heinlein et al., 2017; Fairchild & Van Hell, 2017; Fernández Fuertes et al., 2019, 2025; Potter et al., 2019; Van Hell et al., 2015, 2018).

### 2.2. The Processing of Linguistic Codeswitching: Directionality of the Switch

Linguistic codeswitching consists of “the ability to alternate between languages in an unchanged setting, often within the same utterance” (Bullock & Toribio, 2009, p. 2). It has been described as effortless, but psycholinguistic research has shown that it also involves measurable effort, reflected in longer processing times, especially when compared to single language comprehension (e.g., Kaushanskaya et al., 2023; Moreno et al., 2002; Salig et al., 2024). This effort has been shown to be mitigated when certain factors are at play such as how used the bilingual is to codeswitching (i.e., habitual codeswitchers vs. non-habitual codeswitchers) (e.g., Adamou & Shen, 2019; Valdés Kroff et al., 2017).

Intrasentential codeswitching, the type of codeswitching occurring within the sentence (e.g., *el niño está leyendo el* book for the first time—the kid is reading the book for the first time), entails the integration of the two languages within the same structure, as well as the presence of multiple grammatical categories (e.g., determiners, nouns, verbs, prepositions, etc., as in the previous example) and not only nouns, as it is typically the case in language switching paradigms (Valdés Kroff & Dussias, 2023). Given the differences between intrasentential codeswitching and the unrelated items paradigm, a growing body of research is questioning whether the patterns and mechanisms observed in paradigms where languages are presented in isolation can also be observed or are parallel to those found when the two languages are integrated in the same structure (Van Hell et al., 2015).

Previous research focusing on intrasentential codeswitching with comprehension tasks has mostly examined bilingual adults using behavioral and neuroimaging measures in order to explore the switch costs this kind of codeswitching entails. Most of the time, the comparison has been made between a unilingual structure and a switched structure (e.g., Altarriba et al., 1996; Moreno et al., 2002). Altarriba et al. (1996) use an eye tracking during reading task and observe a higher cognitive cost when there is a switch in the sentence compared to the non-switched counterpart. In the same line, Moreno et al. (2002) use ERPs to test English–Spanish bilingual adults. Their results indicate that these bilinguals perceive switches as unexpected events, and thus, they require higher cognitive load.

Altarriba et al. (1996) and Moreno et al. (2002) explore whether codeswitching entails a switching cost when compared to unilingual structures, but they do not take into account different codeswitching structures to examine whether one directionality is more costly than the other (i.e., switching from the dominant into the non-dominant language or vice versa). More recent studies examining adult bilinguals have considered the two directionalities (e.g., Beatty-Martínez & Dussias, 2019; Bultena et al., 2015; Fairchild & Van Hell, 2017; Fernández Fuertes et al., 2019; Litcofsky & Van Hell, 2017). Litcofsky and Van Hell (2017), for example, explore directionality effects in codeswitching and switching costs with behavioral (self-paced reading task) and EEG techniques (ERPs and time frequency). They compare the two directionalities to unilingual sequences to see whether processing costs can be found due to the switch and to the directionality of the switch (from the weaker to the dominant language). Switches are mid-sentence, between the determiner and the noun, and the sentence continues in the switched language until the end. Litcofsky and Van Hell (2017) find different results based on the type of elicitation technique. Behavioral measures reveal higher processing costs when switching into the dominant language, as found in previous production studies which use isolated item paradigms. Conversely, the measures obtained from the EEG technique point to more cognitive load when switching into the weaker language due to the reorganization or adaptation of the syntactic structure in question. This sentence-level restructuring may be caused by the grammatical requirements of one or both languages that force modifications in the form of adjustments or accommodations (e.g., gender, number, and agreement).

Switching costs and directionality effects in codeswitching have also been explored, to a lesser extent, with data from bilingual children (e.g., Byers-Heinlein et al., 2017; Fernández Fuertes et al., 2025; Gross et al., 2019; Potter et al., 2019). Byers-Heinlein et al. (2017) test 20-month-old children from bilingual communities with a looking-while-listening task. Their aim is to examine whether bilinguals monitor and control their languages during language processing and how language monitoring and control would entail a higher cognitive load when hearing a language switch (p. 9033). They find that intrasentential codeswitching imposes a processing difficulty. When contrasting directionalities, they find that children show higher processing costs when they hear switches into their non-dominant language, similar to Litcofsky and Van Hell (2017). Therefore, previous findings have suggested a link between dominance and processing costs. Using an auditory moving window paradigm, Gross et al. (2019) elicit data from bilingual children who are presented with bilingual and unilingual sentences with both switching directionalities. These bilinguals also show higher processing costs with the switched sequences but, in this case, no directionality effects appear (i.e., regardless of whether the switch happens into the dominant language or into the non-dominant language).

### 2.3. The Processing of Linguistic Codeswitching: Gender Agreement

Some recent studies have combined psycho- and neurolinguistic techniques as well as formal linguistic approaches in order to provide an explanation for the processing patterns resulting from linguistic codeswitching when grammatical gender is involved. Fairchild and Van Hell (2017) and Fernández Fuertes et al. (2019) explore adult bilinguals’ codeswitching patterns in terms of the directionality of the switch and the gender agreement mechanisms involved in determiner phrases (i.e., when the switch happens between the determiner and the noun, as in 1 and 2 above). Fairchild and Van Hell (2017) aim to explore how the two main codeswitching theoretical frameworks (i.e., the Matrix Language Frame, Myers-Scotton, 1993, and the Minimalist Program, MacSwan, 1999) can account for intrasentential codeswitching patterns by eliciting codeswitching data via psycholinguistic techniques. They collect data from adult English-dominant Spanish–English bilinguals by using three picture naming tasks: a bare picture task, a determiner–noun picture task and a sentence context task. Participants are asked to name each picture using a unilingual determiner phrase in English (the house) or Spanish (*la casa*) or either an English determiner switch (the *casa*) or a Spanish determiner switch (*la/el* house). By analyzing the reaction times and accuracy rates of the participants’ production, the researchers observe that bilinguals are slower and less accurate when producing Spanish determiner switches (*la/el* house). They also explore the gender agreement mechanisms involved in order to see whether participants adhere to the masculine as a default strategy, as can be observed in other studies (e.g., Denbaum & de Prada Pérez, 2021; Valdés Kroff, 2016; Valenzuela et al., 2012), but they find that only 7.2% use this strategy.

Following Minimalist premises in codeswitching and considering the role played by formal features, in line with previous studies such as that by Liceras et al. (2008), Fernández Fuertes et al. (2019) use an eye tracking during reading experiment to collect data from L1 Spanish–L2 English bilingual adults. They aim to explain how the representation of Spanish grammatical gender features plays a role in how bilinguals process intrasentential codeswitching. They find higher processing costs when the determiner is in Spanish and the noun in English (*la/el* house), something they attribute to gender agreement mechanisms: as the noun is in English, the Spanish translation equivalent may be retrieved, and, if so, this may trigger gender agreement operations between the Spanish determiner and the English noun. That is why English determiner switches (the *casa*) seem to be easier to process for the bilingual speaker, because no gender agreement operations can be triggered. They also observe that these bilinguals show fewer processing costs with Spanish determiner switches where gender agreement is successful (i.e., congruent structures such as *la* house—where the gender of the determiner matches the gender of the translation equivalent of the English noun). Thus, the authors point to grammatical gender features as the ones guiding bilingual processing in switched determiner phrases.

Fernández Fuertes et al. (2025) aim to explain mechanisms such as activation and inhibition in bilingual structures in order to capture the interaction between processing and feature valuation considering the role of grammatical features. The researchers propose a scale of processing difficulty for the three possibilities that can result in the case of Spanish determiner switches (as in 2): activation proper (*la* house); activation by default (*el* house); and local inhibition (*la/el* house). Activation proper would take place in gender congruent structures, as in 2a, where there is (1) retrieval and activation of the Spanish noun (window > *ventana*), (2) its classification as a Spanish noun (*ventana* = feminine), and (3) the implementation of the gender feature valuation process (*la*_fem_ window *_ventana_* − _SP fem_). These three steps may trigger a slowdown in processing in comparison to (i) activation by default (2c), where there is a relaxation of the gender agreement requirements so the masculine default determiner can be followed by either a masculine or a feminine noun, or to (ii) local inhibition, where there is suppression of the Spanish translation equivalent, so no gender agreement takes place. The researchers test this scale with online and offline data (eye tracking during reading and acceptability judgments) from a group of L1 Spanish–L2 English bilingual children. They find that offline and online data go hand in hand, as faster processing times are linked to higher ratings. Their L1 Spanish bilingual children show lower processing costs and a preference for structures where English provides the determiner (the *casa*), as they are the most economical in terms of processing. In Spanish determiner switches (*la/el* house), these bilinguals show lower processing costs in structures where there is gender congruency (*la* house, *el* book, as in 2a) in opposition to those where there is not (*el* house, *la* book, as in 2b). This is attributed to how rooted Spanish grammatical features are in these speakers’ minds. Fernández Fuertes et al. (2025) conclude that formal features could explain how the relation between cognitive mechanisms such as activation and inhibition proceed in bilingual processing.

What all these previous works on bilingual processing show is that the two languages seem to always be active with some kind of interaction occurring, even when only one is being used. This triggers cognitive control mechanisms like inhibition to manage competition between the two languages. Interaction can lead to processing costs, especially when switching between languages, with factors such as language dominance and task type influencing these costs. The potential processing costs that could be associated with codeswitching are argued to vary depending on experience and context. In this respect, research has shown that the direction of the switch and grammatical features, such as gender agreement, can affect processing, with some structures being easier when they align with bilinguals’ dominant language or involve fewer grammatical adjustments.

## 3. The Current Study

We follow Fernández Fuertes et al.’s (2025) premises to understand the connection established between the properties of the two languages of the bilingual and the different cognitive processes involved during processing. In order to do so, we use linguistic codeswitching as a linguistic context where the respective grammars of the bilingual are required to be activated. We aim to explore whether the status that Spanish has in the mind of these bilinguals (balanced vs. English-dominant) has an impact on how they process the directionality of the switch and the different gender agreement mechanisms in Spanish determiner switches and, thus, how their mental representation of formal features is linked to the diverse cognitive mechanisms involved in bilingual processing. Given that previous works have shown dominance effects, we would also consider different dominance groups measured using the BLP (Birdsong et al., 2012), which is a well-known and well-established instrument that has been tested for reliability (e.g., Amengual, 2026; Olson, 2023). The BLP contains an introductory section for collecting biographical information and four modules designed to assess different dimensions of dominance (language history, language use, language proficiency, and language attitudes). The BLP has been translated into dozens of languages, and the instrument is available in a variety of language pairs (Birdsong et al., 2012).

This study complements the one by Fernández Fuertes et al.’s (2025) by considering a bilingual population with a different linguistic profile (simultaneous English–Spanish bilingual children) and by addressing the role played by language dominance.

To address these issues, the following research questions are proposed:

1. Which patterns appear when simultaneous bilinguals process linguistic codeswitching involving grammatical gender features? In this case, we examine both the directionality of the switch and gender agreement mechanisms as follows:
  1.1. Which patterns can be found in terms of directionality? The aim is to examine which structures are processed faster by these simultaneous bilinguals: structures where no gender agreement mechanisms are enforced between the English determiner and Spanish noun (i.e., English determiner switches as in example (1a) above) or structures where the determiner is in Spanish and where gender agreement may occur (i.e., Spanish determiner switches as in example (1b) above). If the economy of derivation is guiding these bilinguals’ processing patterns, the prediction would be for English determiner switches having fewer processing costs.
  1.2. When these simultaneous bilinguals are presented with Spanish determiner switches (as in example (2) above), which gender agreement strategy is processed faster? In this case, we examine whether these bilinguals impose activation proper (i.e., where agreement between the Spanish determiner and the translation equivalent of the English noun occurs, as in example (2a) above) or not, as in example (2b) above, or whether they process faster switches in which activation by default takes place, (i.e., masculine as default, as in example (2c) above). If, for these bilinguals, valuing grammatical gender features is required, then activation proper leading to gender congruent switches would not take longer to process when compared to non-congruent switches.
2. Do these patterns differ in terms of the participants’ language dominance (i.e., balanced bilinguals vs. English-dominant bilinguals)? If switching from the dominant language into the non-dominant language shapes bilinguals’ processing patterns, this would translate into a different processing behavior between the two dominance groups.

## 4. Methods

### 4.1. Participants

We examine data from English–Spanish simultaneous bilingual children (n = 36)^2^ who have been born and brought up using the two languages from birth and also in a social environment where conversational codeswitching is used. They come from two schools from two language-contact areas: Gibraltar (UK) and Sotogrande (Spain). Gibraltar is an overseas British territory located in southern Spain, where English is the official language and Spanish is in constant contact due to its social and historical presence in Gibraltar (e.g., Levey, 2008, 2022; Loureiro-Porto & Suárez-Gómez, 2017; Weston, 2013; Seoane et al., 2024). Sotogrande is located in Cádiz, Spain, and it can be considered an “English pocket” due to the strong English community that lives there. Their bilingualism may start individually in the home context, but it has become social, as both English and Spanish, as well as other languages, are used within the community. The children from Sotogrande participating in this study were taking classes in an international school where English is the main language of instruction and where children with other nationalities also study.

Participants have been divided into two groups, balanced bilinguals and English-dominant bilinguals, as per the dominance scores obtained via the BLP. Balanced bilinguals are considered to be those scoring between −50 and +50 points, while English-dominant bilinguals are the ones scoring from +50 points on. A summary of the participants appears in Table 1.

### 4.2. The Experiment

Online processing data have been collected via an eye tracking during reading experiment, which consists of 156 sentences organized into 48 experimental items, 54 distractors, and 54 fillers.^3^

Each experimental item is composed of two Spanish nouns, masculine and feminine, and their English translation equivalents (e.g., *libro*_Masc_—book; *ventana*_Fem_—window). The Spanish nouns are preceded by an English determiner, while the English nouns are preceded by a Spanish determiner. In the latter case, the structures have been manipulated to have one congruent form, where the Spanish determiner agrees in gender with the Spanish translation equivalent of the English noun (e.g., *el*_Masc_ book _SP Masc_; *la*_Fem_ window _SP Fem_), and a non-congruent form, where there is no agreement (e.g., *la*_Fem_ book _SP Masc_; *el*_Masc_ window _SP Fem_). In the case of the English determiner + Spanish noun sequences, as no gender agreement can be imposed, only one form of each condition is included (an English determiner with a Spanish feminine noun, DF, or an English determiner with a Spanish masculine noun, DM). Thus, each experimental item has six experimental conditions represented by six sentences, as in Table 2. However, each participant only reads one sentence per experimental item, as there are six different lists.

As in Table 2, each sentence consists of a target determiner phrase which is preceded by four pre-target words and two to four post-target words. The language of the post-target words depends on the language of the target noun; that is, if the language of the noun is Spanish, the post-target words are in Spanish. The target words always occupy the direct object position. Target nouns are inanimate and concrete, and they do not involve body parts or cognates. Also, nouns in either language do not begin with a vowel, and English nouns do not begin with a <l>.

We have used three databases to calculate frequency of the target nouns: EsPal database (Duchon et al., 2013) and the SUBTLEX-ESP database (Cuetos et al., 2011) and SUBTLEXus database (Brysbaert & New, 2009). Frequency has been controlled for both English and Spanish experimental nouns. An independent two-tailed *t*-test comparing the frequency of Spanish nouns in terms of gender (masculine versus feminine) indicates no significant differences (*t*(94) = 0.959, *p* = 0.345). Another independent two-tailed *t*-test has been performed to compare the frequency of English nouns with masculine Spanish translation equivalents (e.g., book) and that of English nouns with feminine Spanish translation equivalents (e.g., window). The comparison does not render significant differences (*t*(94) = −1.144, *p* = 0.256).

This experiment also includes 54 fillers and 54 distractors to switch the attention of the participants away from the experimental sentences. Fillers are monolingual sentences (half in Spanish and half in English) which contain a noun–noun compound word placed in the initial, mid, or final position of the sentence (example in English: the little girls have a beautiful coffee cup). Distractors, on the other hand, are bilingual sentences containing a switch between the subject and the predicate. Half of the distractors have a Spanish subject and an English predicate (e.g., *el payaso*_the clown_ has a very big red nose), while the other half contains the opposite directionality (e.g., the kids *llegan a la escuela en bicicleta* _get to school by bike_). Half of the fillers and half of the distractors contain a comprehension question to make sure that participants are paying attention to the task. All fillers and distractors contained between 8 and 10 words—similar length to that of the experimental sentences.

Before the experimental blocks, participants read 9 practice sentences which contain codeswitching at different grammatical points that do not coincide with those found in the distractors nor in the experimental sentences. Neither the practice sentences, the fillers, nor the distractors include words that appear in the experimental sentences.

### 4.3. Data Collection Procedure

Participants were tested in a quiet room in an institutional setting in Gibraltar and Sotogrande. Eye movement data were collected with an EyeLink Portable Duo head free-to-move eye tracker, which samples eye movements at 1000 Hz. They performed the experiment at 60 inches from a 17-inch laptop screen.

Before carrying out the task, the participants’ parents had been informed about the procedure, and they had signed a consent form. They also completed an adapted version of the BLP (Birdsong et al., 2012) on Microsoft Forms, where they gave information about their children’s language practices.

Participants were asked to complete the task in silence, at their own pace, after they had performed a 3-point calibration, and the average error was under 0.5°.

The task consists of a practice set and four blocks balanced in terms of experimental (n = 12), filler (n = 13), and distractor (n = 14) items, resulting in 39 sentences per block. After each block, they could take a break. If they moved or the break was too long, participants were recalibrated before starting the new block.

### 4.4. Data Codification and Analysis Procedure

The aim of the present study is to examine the processing relationship that can be established between the determiner and the noun, that is why each experimental sentence, as described above in Section 4.2., has been divided into four interest areas, a pre-target area, 2 target areas (determiner and noun), and a post-target area, as in Table 3.

The determiner is a functional category, very short in length, and, as it is a very frequent category both in English and in Spanish, it tends to be skipped when reading (Conklin et al., 2018; Godfroid, 2020; Serrano-Carot & Angele, 2026). That is why we have decided to include the three letters and the space prior to the determiner as part of the target area of the determiner. The reasoning behind this decision comes from the consensus that short words are identified during the fixation of prior words (Rayner, 2009).

For this study, several measures have been considered in the analysis: (i) In the case of the noun target region, as we are interested in the relationship between the determiner and the noun in terms of operations triggered by grammatical gender features, we have used the first fixation duration measure to test the duration of the first fixation on the noun target region, as in 3; (ii) for the determiner target region, we have analyzed whether there have been regressions into that region, as in 4.^4^


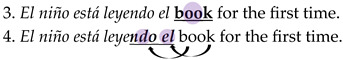


Prior to the statistical analyses presented in Section 4, the data obtained from the first fixation duration measure in milliseconds (ms) have been screened and cleaned for outliers. For an item to be included in the analysis, it had to be fixated for less than 1200 ms. Later, these data were log-transformed (Winter, 2020).

## 5. Eye Tracking During Reading Results

We have run six models: three Linear Mixed Effects models with the first fixation duration data (Models S1, S3 and S5) and three Generalized Linear Mixed models with the regression data (Models S2, S4 and S6). All of them have been run using the lme4 package in R (Bates et al., 2015) to analyze the directionality of the switch (Models S1 and S2) and the gender agreement mechanisms (Models S3–S6). In Models S1 and S2, we have incorporated both English determiner and Spanish determiner switches to compare the patterns in terms of directionality. These models contain directionality (English determiner vs. Spanish determiner) and dominance group (English-dominant vs. balanced as per BLP scores) as fixed factors and are controlled for participants as a random factor. Models S3–S6 only include the Spanish determiner switches, as the focus is on gender agreement mechanisms. Models S3 and S4 include the congruency of the switch (gender congruent vs. non-congruent switches) and the dominance group (English-dominant vs. balanced as per BLP scores) as fixed factors and are controlled for participants as a random factor. Models S5 and S6 include all conditions in the case of Spanish determiner switches (FF, FM, MF, and MM; see Table 2) and the dominance groups (English-dominant vs. balanced as per BLP scores) as fixed factors and are controlled for participants as a random factor. Post hoc analyses have been run using the Bonferroni adjustment with the emmeans function (Lenth, 2022) to further investigate interactions.

### 5.1. Directionality of the Switch^5^

Results from the directionality of the switch in terms of the first fixation duration measure (Model S1) appear in Table 4. In this case, the comparison is between Spanish determiner switches (*la* house/*el* book) and English determiner switches (the *casa/libro*).

**Figure 1:**
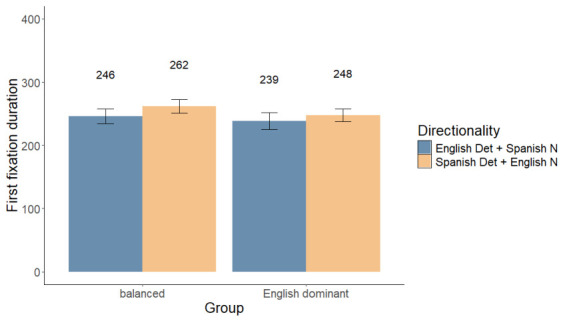
English determiner switches (the *casa*) vs. Spanish determiner switches (*la* house).^6^ Note: The error bars represent the 95% confidence intervals around the mean.

When it comes to the directionality of the switch, Model S1 using first fixation duration data shows no effect in terms of directionality (*beta* = 0.02, *95% CI* [−0.04, 0.08], *t* = 0.69, *p* = 0.48; *Std. beta* = 0.05, *95% CI* [−0.09, 0.19]) or group (*beta* = −0.03, *95% CI* [−0.16, 0.09], *t* = −0.49, *p* = 0.62; *Std. beta* = −0.07, *95% CI* [−0.37, 0.23]) and no interaction between group and directionality (*beta* = 0.02, *95% CI* [−0.07, 0.10], *t* = 0.38, *p* = 0.70; *Std. beta* = 0.04, *95% CI* [−0.16, 0.24]). This means that both balanced and English-dominant groups show similar processing patterns in terms of directionality and that both participant groups behave similarly when processing the two directionalities, as shown in Figure 1.

When analyzing regressions, results from Model S2 are presented in Table 5. These are the comparisons of the proportions of regressions made to the determiner target region in terms of directionality; that is, the rate in which there have been regressions to the determiner target region in English determiner switches (the *casa*) and Spanish determiner switches (*la/el* house).

**Figure 2:**
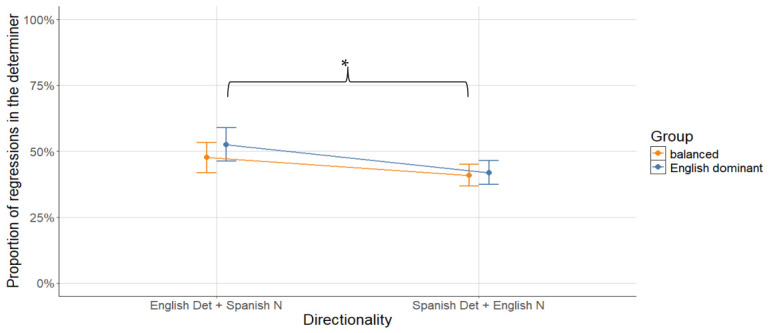
Proportion of regression to the determiner in terms of directionality (*the* casa vs. *la/el* house). Note: * indicates significant results (<0.05).

As presented in Table 5, there is no interaction between group and directionality (*beta* = −0.16, *95% CI* [−0.61, 0.29], z = −0.69, *p* = 0.48; *Std. beta* = −0.16, *95% CI* [−0.61, 0.29]) and no effect of group (*beta* = 0.20, *95% CI* [−0.41, 0.81], z = 0.65, *p* = 0.51; *Std. beta* = 0.20, *95% CI* [−0.41, 0.81]), but there is an effect of directionality (*beta* = −0.32, *95% CI* [−0.63, −0.01], z = −2.02, *p* = 0.04; *Std. beta* = −0.32, *95% CI* [−0.63, −0.01]). This indicates that both groups show the same performance in terms of directionality regardless of their language dominance. As illustrated in Figure 2 and observed in the post hoc tests, both groups show a higher regression rate when presented with English determiner switches; that is, they come back more to the English determiner (the *casa*) than to the Spanish determiner (*la/el* house) (balanced bilinguals: *p* = 0.04; English-dominant bilinguals: *p* = 0.004).

To summarize, as for the directionality of the switch, both the first fixation duration measure and the regression measure indicate no differences between groups. This means that, regardless of their language dominance, both balanced and English-dominant bilinguals present the same patterns. In the case of the first fixation duration measure, there seem to be no processing costs when comparing across directionalities. However, the regression measure points to processing costs when switching from English into Spanish. This difference in the way both groups of speakers seem to process Spanish determiner switches (*la* house) versus English determiner switches (the *ventana*) could be suggestive of English determiner switches blocking gender valuation. Even if the Spanish noun requires its gender features to be valuated, regression to the English noun to perform this operation results in an impossibility to do so, as English determiners bear no gender features. In the case of Spanish determiner switches, since the English noun bears no gender, there is no need to regress to the Spanish determiner to perform feature valuation.

### 5.2. Gender Agreement Mechanisms

Results regarding the contrast in terms of gender congruency in the case of the first fixation duration measure (Model S3) are presented in Table 6. The comparison has been made between gender congruent switches (*la*_fem_ house _SP fem_/*el*_masc_ book _SP masc_), where there is a match between the gender of the Spanish determiner and the gender of the translation equivalent of the English noun, and gender non-congruent switches (*el*_masc_ house _SP fem_/*la*_fem_ book _SP masc_), where there is a mismatch between the gender of the Spanish determiner and the gender of the Spanish translation equivalent of the English noun.

Results from Model S3, presented in Table 6, indicate no effects in terms of group (*beta* = −0.03, *95% CI* [−0.15, 0.10], *t* = −0.40, *p* = 0.69; *Std. beta* = −0.06, *95% CI* [−0.35, 0.23]) or gender congruency (*beta* = 0.04, *95% CI* [−0.03, 0.11], *t* = 1.10, *p* = 0.27; *Std. beta* = 0.09, *95% CI* [−0.07, 0.25]) and no interaction between group and gender congruency (*beta* = 0.02, *95% CI* [−0.08, 0.12], *t* = 0.35, *p* = 0.730; *Std. beta* = 0.04, *95% CI* [−0.19, 0.28]). This means that both groups present the same performance regardless of their language dominance scores and regardless of the type of gender agreement.

**Figure 3:**
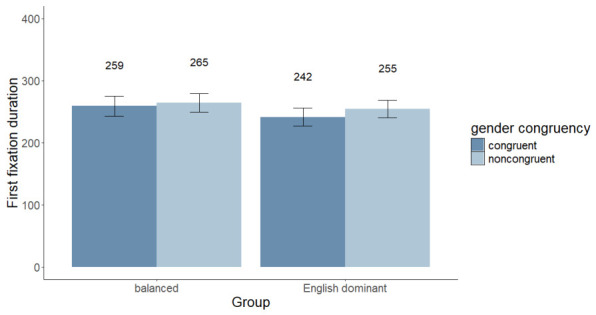
Gender congruent switches (*la* door) vs. gender non-congruent switches (*la* dress). Note: The error bars represent the 95% confidence intervals around the mean.

Figure 3 displays very similar durations of the first fixations in both congruent and non-congruent structures. This means that neither the balanced nor the English-dominant bilinguals show processing costs towards sequences where there is gender agreement or towards those where there is no gender agreement between the Spanish determiner and the Spanish translation equivalent of the English noun.

The same results have been found in Model S4 in terms of the regression measure, as presented in Table 7.

**Figure 4:**
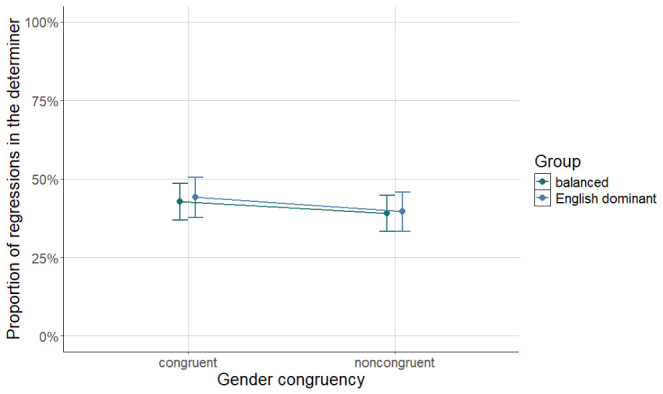
Proportion of regressions to the determiner in terms of gender congruency (*la* door vs. *la* dress).

Model S4 indicates no effect of group (*beta* = 0.06, *95% CI* [−0.61, 0.73], *z* = 0.18 *p* = 0.85; *Std. beta* = 0.06, *95% CI* [−0.61, 0.73]) nor of gender congruency (*beta* = −0.17, *95% CI* [−0.54, 0.19], *z* = 0.18; *p* = 0.355; *Std. beta* = −0.17, *95% CI* [−0.54, 0.19]) and no interaction between group and gender congruency (beta = *−0.06*, *95% CI* [−0.60, 0.48], *z* = −0.20, *p* = 0.83; *Std. beta* = −0.06, *95% CI* [−0.60, 0.48]). As represented in Figure 4, both groups have very similar regression rates to both gender congruent (*la* door) and gender non-congruent switches (*la* dress), indicating no higher processing costs for either structure.

**Figure 5:**
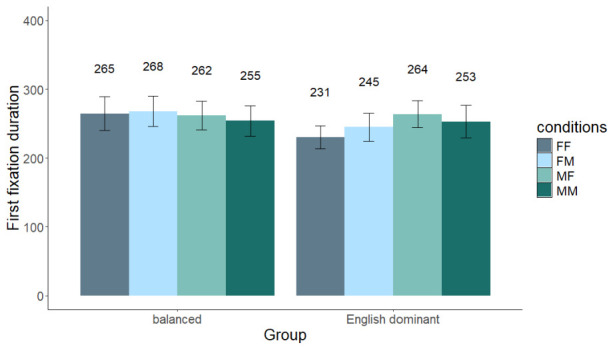
All conditions of the Spanish determiner switches as of the first fixation duration measure. Note: FF = Spanish feminine Det + feminine equivalent noun; FM = Spanish feminine Det + masculine equivalent noun; MF = Spanish masculine Det + feminine equivalent noun; and MM = Spanish masculine Det + masculine equivalent noun.

**Figure 6:**
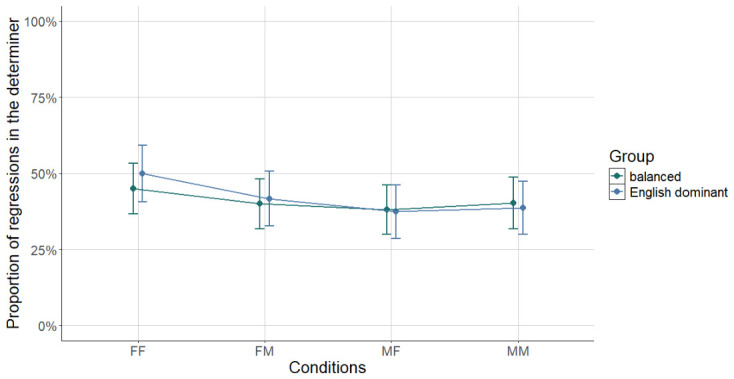
Proportion of regressions to the determiner in terms of all the Spanish determiner conditions. Note: FF = Spanish feminine Det + feminine equivalent noun; FM = Spanish feminine Det + masculine equivalent noun; MF = Spanish masculine Det + feminine equivalent noun; and MM = Spanish masculine Det + masculine equivalent noun.

Further analyses have been carried out to explore the differences across conditions within each group and across groups to determine whether there is an effect of the masculine as the default option. Neither the data of the first fixation duration measure (Table 8 and Figure 5) nor of the regression measure (Table 9 and Figure 6) indicate differences across conditions nor across groups.

To summarize, the results in terms of gender agreement mechanisms (gender congruent, gender non-congruent, and masculine as default), using both the first fixation duration measure and the regression measure, indicate no processing costs for either condition. This points to all three mechanisms being processed similarly by both bilingual groups. In addition, no differences across groups have been found, even though they have different profiles based on their language dominance.

## 6. Discussion and Conclusions

Following previous studies, which have combined formal linguistic and psycholinguistic approaches (e.g., Belazi et al., 1994; Fairchild & Van Hell, 2017; Fernández Fuertes et al., 2019, 2025; MacSwan, 2000; Toribio, 2011), the present study has aimed to explore the connection between the properties of the two languages of the bilingual and the different cognitive processes that are set in motion when the two languages are forced to be activated, i.e., in linguistic codeswitching. In particular, this has been explored within the determiner phrase, when there is a language switch between the determiner and the noun. With a focus on how formal features, namely, grammatical gender, can guide these cognitive processes, we have formally examined the directionality of the switch (i.e., *la/el* house vs. the *casa*) and the gender agreement mechanisms in Spanish determiner switches (i.e., gender congruent, *la* house; gender non-congruent, *la* book; and masculine as default, *el* house).

We have elicited data from simultaneous bilingual children from two English–Spanish bilingual communities. Although data from children are increasingly being targeted in recent investigations on codeswitching (e.g., Byers-Heinlein et al., 2017; Fernández Fuertes et al., 2025; Gross et al., 2019; Potter et al., 2019), not much research has been done that takes into account different groups that vary in terms of their language dominance. In this case, these simultaneous bilingual children have been grouped in terms of their dominance scores as measured by the BLP (Birdsong et al., 2012). This way, we can determine whether language dominance plays a role, or rather, the language properties of the two languages involved in the switch could be argued to guide bilingual processing. To address how the properties of the two languages are mentally represented and how these mental representations may shape these bilinguals’ processing mechanisms, we collected data using an eye tracking during reading experiment. We have used the first fixation duration measure on the noun target region and the rate of regressions to the determiner target region to explore the processing relationship between the determiner and the noun.

In terms of the directionality of the switch, the contrast has been made between a structure where gender agreement may be enforced (Spanish determiner switches, *la/el* house) and a structure where there is no such enforcement due to the lack of grammatical gender in English (English determiner switches, the *casa*). In the present study, these simultaneous bilinguals do not show significant differences when comparing the way they process the two directionalities in the case of the first fixation duration measure, and this is true regardless of their dominance group. This suggests that there are no switch costs when processing codeswitching within the determiner phrase with either directionality.

However, when it comes to the regression rate, both groups have done more regressions to the English determiner after reading the Spanish noun (i.e., the *casa*). This result may be more in line with the profile of the English-dominant bilinguals, because the switch is into their non-dominant language, Spanish, as it has been observed in previous studies (e.g., Byers-Heinlein et al., 2017; Litcofsky & Van Hell, 2017). Nonetheless, the same pattern is found in the regression data from the balanced bilingual group. In this case, the same reasoning cannot be behind their higher processing costs in switches into their non-dominant language, as both English and Spanish are equally dominant for them according to their BLP scores (Birdsong et al., 2012). In fact, the expected outcome for the balanced bilingual children would have been no differences in terms of regression rates to either the English or the Spanish determiner. Given that both participant groups show the same pattern, we would like to argue that one potential explanation would be that it is not so much a matter of switching into the non-dominant language but rather a question of not establishing the gender agreement between the determiner and the noun. That is, given that this pattern is found for the two dominance groups that we analyzed, an alternative explanation could be that both bilingual groups resort to a more economic derivation that, in turn, does not delay processing. If regressions are interpreted as re-analyses (Wilcox et al., 2024), a higher proportion of regressions to the English determiner could suggest that both bilingual groups are regressing to the English determiner to enable some sort of valuation mechanism relative to the Spanish noun. That is, although the Spanish noun requires its gender features to be valued, attempting to carry out this operation via an English determiner fails, given that English determiners do not have gender features. If these bilinguals regressed to the Spanish determiner in Spanish determiner switches (*la/el* house), they could be regressing to check gender features against the Spanish determiner if the English noun is classified as a Spanish noun. This, indeed, would entail higher processing costs, as seen in Fernández Fuertes et al. (2019, 2025) for L1 Spanish–L2 English bilinguals. However, the participants in this study are doing the opposite. Thus, this may suggest that neither group wants to enforce gender agreement when this could be a possibility, that is, in the case of Spanish determiner switches.

This idea of not enforcing gender agreement receives further support with the results obtained in terms of gender agreement mechanisms in Spanish determiner switches. In this case, we compare structures where gender agreement may be enforced: gender congruent switches (*la* house), where there is a match between the gender of the determiner and the one for the Spanish translation equivalent of the English noun (house > *casa*), and gender non-congruent switches (*la* book), where there is no such match. In this comparison, both the first fixation duration measure on the noun and the regressions to the determiner point to no processing costs for either structure. With feature valuation in mind, all potential differences across structures seem to be neutralized. That is, if “*la* book” (FM) and “*la* window”(FF) and “*el* book” (MM) and “*el* window” (MF) are all processed similarly (which is what the lack of significance could be argued to point at), what we would like to suggest is that these speakers do not seem to be classifying the English noun as a Spanish noun, and therefore, gender features are irrelevant in this respect. If significant differences were found, this could suggest that some sort of classification is enforced, which would, in turn, trigger some sort of gender feature valuation. Taken together, the faster processing attributed to English determiner switches and the lack of differences in the case of Spanish determiner switches between congruent and non-congruent switches lead us to propose an explanation based on the most economical option in the hierarchy of processing difficulty proposed by Fernández Fuertes et al. (2025): local inhibition. That is the fact that, in the case of Spanish switches, both congruent and non-congruent switches are processed similarly may be suggestive of a suppression of the retrieval of the Spanish translation equivalent, so no gender agreement takes place. Local inhibition would be the easiest structure to process, as it does not involve the activation of the translation equivalent, the classification of the Spanish noun, nor the implementation of gender agreement. This is what the results from the bilinguals from our study could be pointing to. That is, for them, it seems to be easier not to enforce gender agreement mechanisms, as they need not go through any of those steps (activation, classification, and feature implementation).^7^ This differs from what has been shown for speakers for whom Spanish is the L1 and English is the L2, where a preference for congruent switches is clearly seen in both online and offline processing (Fernández Fuertes et al., 2025).

To have a more complete picture of how these bilinguals process gender agreement mechanisms, we have also compared all conditions of the Spanish determiner switches within each group and across groups. This way, we are able to explore whether the masculine as a default strategy is used, as it has been reported in previous research with production and judgment data (e.g., Denbaum & de Prada Pérez, 2021; Valdés Kroff, 2016; Valenzuela et al., 2012). However, as in Fairchild and Van Hell (2017), these two bilingual groups do not seem to resort to this type of strategy, at least when it comes to processing. Again, this points to not enforcing gender agreement as the easiest processing option.

Interestingly, as with directionality, the two bilingual groups appear to exhibit highly similar processing behavior, regardless of their dominance scores. Neither the contrast in gender congruency nor the contrast across conditions reveals dominance group-based differences.

Although data from objective measures of proficiency and processing efficiency could provide a more refined picture, the processing data that we have obtained in combination with BLP dominance scores allows us to draw two main conclusions. The first one is that these bilinguals, regardless of their language dominance, do not seem to show asymmetrical processing costs when presented with codeswitching within the determiner phrase in either directionality. This lack of asymmetrical switching costs is in line with previous studies, which have used comprehension tasks (Declerck & Grainger, 2017). When processing costs appear, as per their regressions to the English determiner (the *casa*), a possible explanation would be to resort to the linguistic properties of the two languages involved in the switch. In this respect, it could be argued that what is at stake is the role played by formal features and how these are mentally represented for these bilinguals. That is, they do not show processing costs in Spanish determiner switches because the most economical option for them in terms of processing seems to be not enforcing gender agreement. Thus, formal features may be behind these bilinguals’ processing performance. It is also important to point out that we are dealing with bilinguals who come from bilingual communities where English and Spanish are in constant contact, so conversational codeswitching is a common practice. Thus, as seen in previous research, their linguistic experience with this practice may be a factor that mitigates the processing effort that codeswitching can entail for other types of bilinguals (e.g., Adamou & Shen, 2019; Valdés Kroff et al., 2017).

The second conclusion is that language dominance, as measured by subjective tools such as the BLP (Birdsong et al., 2012), may not be giving us the full picture, as indeed, bilinguals with different dominance profiles need not differ in their processing strategies. We have tested two groups which differ in terms of their language dominance, i.e., balanced and English-dominant, but they have shown the same patterns in terms of directionality and gender agreement mechanisms, and this has been so for both measures: first fixation duration and regressions. Thus, to explain bilinguals’ processing, not only do subjective tools measuring language dominance need to be taken into account, but also information pointing to the mental representation of the properties of the two languages of the bilingual in order to have a more complete view of the bilingual mind.

Finally, we believe that we need to consider that these bilinguals are still children, so language experience may play a role in how formal features are represented in their minds and how they guide their processing behavior. In this respect, future research may consider comparing these bilingual children to balanced and English-dominant bilingual adults from the same communities. To further explore processing mechanisms, these results could be compared to those obtained using other online measures like reaction times or offline measures like acceptability judgments. Likewise, experiments that involve visual and auditory stimuli, such as the visual world paradigm, would allow us to further explore and contribute to providing a more complete picture of the multifaceted nature of bilingualism.

## Figures and Tables

**Table 1:** Summary of the participants.

Group	Number of Participants	Mean Age (SD)
Balanced	20	12.77 (1.42)
English-dominant	16	13.50 (1.40)

**Table 2:** Example of an experimental item.

Condition	Congruency	Pre-Target	Target	Post-Target
MM	Congruent	*El niño está leyendo*	*el* book	for the first time
FF	Congruent	*El señor está arreglando*	*la* window	with a hammer
MF	Non-congruent	*El señor está arreglando*	*el* window	with a hammer
FM	Non-congruent	*El niño está leyendo*	*la* book	for the first time
DM	English Det	The boy is reading	the *libro*	*por primera vez*
DF	English Det	The man is fixing	the *ventana*	*con un martillo*

Note: MM = Spanish masculine Det + masculine equivalent noun; FF = Spanish feminine Det + feminine equivalent noun; MF = Spanish masculine Det + feminine equivalent noun; FM = Spanish feminine Det + masculine equivalent noun; DM = English Det + Spanish masculine noun; and DF = English Det + Spanish feminine noun.

**Table 3:** Interest areas for each experimental sentence.

Pre-Target Area	Target: Determiner	Target: Noun	Post-Target
*El niño está leye*	*ndo el*	book	for the first time
The man is fix	ing the	*ventana*	*con un martillo*

**Table 4:** Summary of Model S1 regarding the results of the directionality of the switch with first fixation duration data.

Measure	Model Parameters			
	*b*	*SE*	*t*	*p*
Intercept	5.429	0.042	127.940	<0.001 *
Group (English-dominant)	−0.031	0.06	−0.499	0.62
Directionality (Spanish determiner)	0.020	0.02	0.696	0.48
Group (English-dominant) x directionality (Spanish determiner)	0.016	0.04	0.379	0.70

Note: * indicates significant results (<0.05).

**Table 5:** Summary of Model S2 regarding the regressions to the determiner in the case of directionality of the switch (*la/el* house vs. the *casa*).

Measure	Model Parameters			
	*b*	*SE*	*z*	*p*
Intercept	−0.1126	0.20	−0.539	0.589
Group (English-dominant)	0.2042	0.31	0.657	0.511
Directionality (Spanish determiner)	−0.3178	0.15	−2.025	0.042 *
Group (English-dominant) x directionality (Spanish determiner)	−0.1594	0.23	−0.692	0.489

Note: * indicates significant results (<0.05).

**Table 6:** Summary of Model S3 regarding the results of the gender congruent vs. non-congruent switches in the first fixation duration measure.

Measure	Model Parameters			
	*b*	*SE*	*t*	*p*
Intercept	5.429	0.04	125.539	<0.001 *
Group (English-dominant)	−0.025	0.06	−0.398	0.692
Gender congruency (non-congruent)	0.039	0.03	1.104	0.270
Group (English-dominant) x gender congruency (non-congruent)	0.018	0.05	0.345	0.730

Note: * indicates significant results (<0.05).

**Table 7:** Summary of Model S4 regarding the regressions to the determiner in terms of the gender congruent vs. non-congruent comparison.

Measure	Model Parameters			
	*b*	*SE*	*z*	*p*
Intercept	−0.3601	0.22	−1.57	0.11
Group (English-dominant)	0.062	0.34	0.18	0.85
Gender congruency (non-congruent)	−0.173	0.18	0.18	0.35
Group (English-dominant) x gender congruency (non-congruent)	−0.056	0.27	−0.20	0.83

**Table 8:** Summary of Model S5 regarding the first fixation duration measure including all conditions of the Spanish determiner switches.

Measure	Model Parameters			
	*b*	*SE*	*t*	*p*
Intercept	5.442	0.05	107.032	<0.001 *
Group (English-dominant)	−0.069	0.07	−0.936	0.352
FM condition	0.038	0.05	0.741	0.459
MF condition	0.015	0.05	0.300	0.764
MM condition	−0.024	0.05	−0.484	0.628
Group (English-dominant) x FM condition	0.009	0.07	0.131	0.896
Group (English-dominant) x MF condition	0.114	0.07	1.541	0.124
Group (English-dominant) x MM condition	0.086	0.07	1.175	0.240

Note: * indicates significant results (<0.05). MM = Spanish masculine Det + masculine equivalent noun; FF = Spanish feminine Det + feminine equivalent noun; MF = Spanish masculine Det + feminine equivalent noun; and FM = Spanish feminine Det + masculine equivalent noun.

**Table 9:** Summary of Model S6 regarding the regressions in the determiner including all conditions of the Spanish determiner switches.

Measure	Model Parameters			
	*b*	*SE*	*z*	*p*
Intercept	−0.240	0.26	−0.919	0.358
Group (English-dominant)	0.158	0.39	0.406	0.685
FM condition	−0.255	0.26	−0.972	0.331
MF condition	−0.330	0.26	−1.264	0.206
MM condition	−0.251	0.26	−0.945	0.345
Group (English-dominant) x FM condition	−0.099	0.38	−0.258	0.797
Group (English-dominant) x MF condition	−0.205	0.38	−0.529	0.597
Group (English-dominant) x MM condition	−0.172	0.38	−0.443	0.657

Note: MM = Spanish masculine Det + masculine equivalent noun; FF = Spanish feminine Det + feminine equivalent noun; MF = Spanish masculine Det + feminine equivalent noun; and FM = Spanish feminine Det + masculine equivalent noun.

## Data Availability

The original data presented in the study are openly available in the University of Valladolid repository (UVaDOC) at https://doi.org/10.35376/10324/54598.

## Notes

1 Formal features are abstract syntactic properties that drive operations like agreement and movement, and whose valuation and checking determine whether a structure is well-formed (Chomsky, 1995; MacSwan, 2000).

2 We are aware that the sample size (n = 36) can influence the statistical power of the study. In this respect, we would like to point out that we are dealing with participants who come from non-extensive populations (Sotogrande and Gibraltar) and who are not adults. These two factors determine data collection.

3 This type of experiment captures where, when, and for how long readers fixate on different parts of a text (Godfroid, 2020).

4 Analyses using total fixation duration measures have also been conducted. As results align with those of regressions, we report the latter.

5 Full model details can be found in the Supplementary Materials, where information about the fixed and random factors included in each model as well as the model details (i.e., AIC and BIC, residuals, number of participants and number of observations) can be found.

6 All statistical analyses have been done using the log-transformed data, but milliseconds have been used to represent the data as shown in the figures.

7 An anonymous reviewer points that our interpretation of local inhibition rests on “null results” and this could be a limitation of the study because we are making an “inference from absence”. Although we fundamentally agree with this and with the fact that the sample size can influence the statistical power of the study, we believe that the data show a clear pattern: (i) statistically significant differences in favor of English determiner switches; (ii) lack of statistically significant differences in favor of either gender congruent or gender non-congruent Spanish determiner switches. These results are consistent with local inhibition even if it is a pattern which partly comes from statistically non-significant differences, something that might still occur even if the number of participants were to increase.

## Supplementary Materials

The following supporting information can be downloaded at: https://www.mdpi.com/article/10.3390/bs16060923/s1, Model S1: Directionality using the first fixation duration measure; Model S2: Directionality using the regressions into the determiner; Model S3: Gender congruency using the first fixation duration measure on Spanish determiner switches; Model S4: Gender congruency using regressions in the determiner in Spanish determiner switches; Model S5: All Spanish determiner conditions using first fixation duration measure; Model S6: All Spanish determiner con-ditions using regressions in the determiner.

## Abbreviations

The following abbreviations are used in this manuscript:

Masc	Masculine
Fem	Feminine
SP	Spanish
EN	English
Det	Determiner
N	Noun
