# Supplementary material for: Language Contact in Bilingual Brains: Formal Features in the Mental Representation of English–Spanish Bilingual Children
Source: Behav Sci (Basel). 2026 Jun 4;16(6):923. doi: 10.3390/bs16060923 (PMC13295466; doi:10.3390/bs16060923)
Supplement: Supplementary file 1 [file behavsci-16-00923-s001.zip › behavsci-4251971-supplementary.pdf]

Supplementary materials

Model S1: Full model details – directionality using the first fixation duration measure.

We fitted a linear mixed model (estimated using REML and nloptwrap optimizer) to predict log_firstfix with BLP_group and directionality (formula: log_firstfix ~ BLP_group x directionality). The model included Participant as random effect (formula: ~1 | Participant). The model's total explanatory power is moderate (conditional R2 = 0.15) and the part related to the fixed effects alone (marginal R2) is 0.001. The model's intercept, corresponding to BLP_group = balanced and directionality = English Det + Spanish N, is at 5.43 (95% CI [5.34, 5.51], t(1483) = 125.05, p < .001).

Model details:

AIC	BIC	logLik	-2xlog(L)	df.resid
1499.8	1531.6	-743.9	1487.8	1483

Groups	Variance	St. dev
Participants (intercept)	0.024	0.15
Residual	0.151	0.38

# of observations: 1489
Participants: 36

**Measure**	**Model parameters**			
	b	*SE*	*t*	*p*
Intercept	5.429	0.042	127.940	<.001*
Group (English dominant)	-0.031	0.06	-0.499	0.62
Directionality (Spanish determiner)	0.020	0.02	0.696	0.48
Group (English dominant) x directionality (Spanish determiner)	0.016	0.04	0.379	0.70

Note: * indicates significant results (<.05)

Model S2: Full model details – directionality using the regressions into the determiner.

We fitted a logistic mixed model (estimated using ML and Nelder-Mead optimizer) to predict regressions_in with BLP_group and directionality (formula: regressions_in ~ BLP_group x directionality). The model included Participant as random effect (formula:~1 | Participant). The model's total explanatory power is moderate (conditional R2 = 0.15) and the part related to the fixed effects alone (marginal R2) is 0.009. The model's intercept, corresponding to BLP_group = balanced and directionality = English Det + Spanish N, is at -0.11 (95% CI [-0.52, 0.30], p = 0.590).

Model details:

AIC	BIC	logLik	-2xlog(L)	df.resid
1990.9	2017.6	-990.5	1980.9	1529

Groups	Variance	St. dev
Participants (intercept)	0.552	0.74

# of observations: 1534
Participants: 36

Measure	Model parameters			
	b	*SE*	*z*	*p*
Intercept	-0.1126	0.20	-0.539	0.589
Group (English dominant)	0.2042	0.31	0.657	0.511
Directionality (Spanish determiner)	-0.3178	0.15	-2.025	0.042*
Group (English dominant) x directionality (Spanish determiner)	-0.1594	0.23	-0.692	0.489

Note: * indicates significant results (<.05)

Model S3: Full model details – gender congruency using the first fixation duration measure on Spanish determiner switches

We fitted a linear mixed model (estimated using ML and nloptwrap optimizer) to predict log_firstfix with BLP_group and AC (formula: log_firstfix ~ BLP_group x AC). The model included Participant as random effect (formula: ~1 | Participant). The model's total explanatory power is weak (conditional R2 = 0.13) andthe part related to the fixed effects alone (marginal R2) is 0.003. The model's intercept, corresponding to BLP_group = balanced and AC = English, is at 5.43 (95% CI [5.35, 5.51], t(968) = 125.54, p < .001).

Model details:

AIC	BIC	logLik	-2xlog(L)	df.resid
1079.1	1108.4	-533.5	1067.1	968

Groups	Variance	St. dev
Participants (intercept)	0.023	0.15
Residual	0.165	0.40

# of observations: 974
Participants: 36

Measure	Model parameters			
	b	*SE*	*t*	*p*
Intercept	5.429	0.04	125.539	<.001*
Group (English dominant)	-0.025	0.06	-0.398	0.692
Gender congruency (non-congruent)	0.039	0.03	1.104	0.270
Group (English dominant) x gender congruency (non-congruent)	0.018	0.05	0.345	0.730

Note: * indicates significant results (<.05)

Model S4: Full model details – gender congruency using regressions in the determiner in Spanish determiner switches

We fitted a logistic mixed model (estimated using ML and Nelder-Mead optimizer) to predict regressions_in with BLP_group and AC (formula: regressions_in ~ BLP_group x AC). The model included Participant as random effect (formula: ~1 | Participant). The model's total explanatory

power is moderate (conditional R2 = 0.18), and the part related to the fixed effects alone (marginal R2) is of 0.002. The model's intercept, corresponding to BLP_group = balanced and AC = congruent, is at -0.36 (95% CI [-0.81, 0.09], p = 0.116).

Model details:

AIC	BIC	logLik	-2xlog(L)	df.resid
1290.6	1108.4	-640.3	1280.6	999

Groups	Variance	St. dev
Participants (intercept)	0.690	0.83

# of observations: 1004
Participants: 36

Measure	Model parameters			
	b	*SE*	*z*	*p*
Intercept	-0.3601	0.22	-1.57	0.11
Group (English dominant)	0.062	0.34	0.18	0.85
Gender congruency (non-congruent)	-0.173	0.18	0.18	0.35
Group (English dominant) x gender congruency (non-congruent)	-0.056	0.27	-0.20	0.83

Model S5: Full model details – all Spanish determiner conditions using first fixation duration measure.

We fitted a linear mixed model (estimated using ML and nloptwrap optimizer) to predict log_firstfix with BLP_group and condition (formula: log_firstfix ~ BLP_group x condition). The model included Participant as random effect (formula: ~1 | Participant). The model's total explanatory power is moderate (conditional R2 = 0.13), and the part related to the fixed effects alone (marginal R2) is 0.007. The model's intercept, corresponding to BLP_group = balanced and condition = FF, is at 5.44 (95% CI [5.34, 5.54], t(964) = 107.03, p < .001).

Model details:

AIC	BIC	logLik	-2xlog(L)	df.resid
1083.0	1131.8	-531.5	1063.0	964

Groups	Variance	St. dev
Participants (intercept)	0.023	0.15
Residual	**0.164**	0.40

# of observations: 974
Participants: 36

Measure	Model parameters			
	b	*SE*	*t*	*p*
Intercept	5.442	0.05	107.032	<.001*
Group (English dominant)	-0.069	0.07	-0.936	0.352
FM condition	0.038	0.05	0.741	0.459

MF condition	0.015	0.05	0.300	0.764
MM condition	-0.024	0.05	-0.484	0.628
Group (English dominant) x FM condition	0.009	0.07	0.131	0.896
Group (English dominant) x MF condition	0.114	0.07	1.541	0.124
Group (English dominant) x MM condition	0.086	0.07	1.175	0.240

Note: * indicates significant results (<.05). MM= Spanish masculine Det + masculine equivalent noun; FF= Spanish feminine Det + feminine equivalent noun; MF= Spanish masculine Det + feminine equivalent noun; and FM= Spanish feminine Det + masculine equivalent noun.

Model S6: Full model details – all Spanish determiner conditions using regressions in the determiner.

We fitted a logistic mixed model (estimated using ML and Nelder-Mead optimizer) to predict regressions_in with BLP_group and condition (formula: regressions_in ~ BLP_group x condition). The model included Participant as random effect (formula: ~1 | Participant). The model's total explanatory power is moderate (conditional R2 = 0.18) and the part related to the fixed effects alone (marginal R2) is 0.006. The model's intercept, corresponding to BLP_group = balanced and condition = FF, is at -0.24 (95% CI [-0.75, 0.27], p = 0.358).

Model details:

AIC	BIC	logLik	-2xlog(L)	df.resid
1295.0	1339.2	-638.5	1277.0	995

Groups	Variance	St. dev
Participants (intercept)	0.689	0.830

# of observations: 1004

Participants: 36

Measure	Model parameters			
	b	*SE*	*z*	*p*
Intercept	-0.240	0.26	-0.919	0.358
Group (English dominant)	0.158	0.39	0.406	0.685
FM condition	-0.255	0.26	-0.972	0.331
MF condition	-0.330	0.26	-1.264	0.206
MM condition	-0.251	0.26	-0.945	0.345
Group (English dominant) x FM condition	-0.099	0.38	-0.258	0.797
Group (English dominant) x MF condition	-0.205	0.38	-0.529	0.597
Group (English dominant) x MM condition	-0.172	0.38	-0.443	0.657

Note: MM= Spanish masculine Det + masculine equivalent noun; FF= Spanish feminine Det + feminine equivalent noun; MF= Spanish masculine Det + feminine equivalent noun; and FM= Spanish feminine Det + masculine equivalent noun.
